# Impact of Glucose-Lowering Agents on the Risk of Cancer in Type 2 Diabetic Patients. The Barcelona Case-Control Study

**DOI:** 10.1371/journal.pone.0079968

**Published:** 2013-11-21

**Authors:** Rafael Simó, Oleguer Plana-Ripoll, Diana Puente, Rosa Morros, Xavier Mundet, Luz M. Vilca, Cristina Hernández, Inmaculada Fuentes, Adriana Procupet, Josep M. Tabernero, Concepción Violán

**Affiliations:** 1 Grup de Recerca en Diabetis i Metabolisme i CIBER de Diabetis i Malalties Metabòliques Associades (CIBERDEM), Institut de Recerca Hospital Vall d'Hebron, Barcelona, Spain; 2 Universitat Autònoma de Barcelona, Bellaterra, Spain; 3 Institut Universitari d'Investigació en Atenció Primària Jordi Gol (IDIAP Jordi Gol), Barcelona, Spain; 4 Section of Epidemiology, Department of Public Health. Aarhus University, Aarhus, Denmark; 5 Institut Català de la Salut, Barcelona, Spain; 6 Servei de Farmacologia Clínica, Hospital Universitari Vall d'Hebron, Barcelona, Spain; 7 Servei Medicina Preventiva i Epidemiologia, Hospital Universitari Vall d'Hebron, Barcelona, Spain; 8 Servei d'Oncologia, Hospital Universitari Vall d'Hebron, Barcelona, Spain; University of Lille Nord de France, France

## Abstract

**Background:**

The aim of the present study is to evaluate the impact of glucose-lowering agents in the risk of cancer in a large type 2 diabetic population.

**Methods:**

A nested case-control study was conducted within a defined cohort (275,164 type 2 diabetic patients attending 16 Primary Health Care Centers of Barcelona). Cases (n = 1,040) comprised those subjects with any cancer diagnosed between 2008 and 2010, registered at the Cancer Registry of Hospital Vall d'Hebron (Barcelona). Three control subjects for each case (n = 3,120) were matched by age, sex, diabetes duration, and geographical area. The treatments analyzed (within 3 years prior to cancer diagnosis) were: insulin glargine, insulin detemir, human insulin, fast-acting insulin and analogues, metformin, sulfonylureas, repaglinide, thiazolidinediones, dipeptidyl peptidase-4 inhibitors, and alpha glucosidase inhibitors. Conditional logistic regressions were used to calculate the risk of cancer associated with the use of each drug adjusted by age, BMI, dose and duration of treatment, alcohol use, smoking habit, and diabetes duration.

**Results:**

No differences were observed between case and control subjects for the proportion, dose or duration of exposure to each treatment. None of the types of insulin and oral agents analyzed showed a significant increase in the risk of cancer. Moreover, no cancer risk was observed when glargine was used alone or in combination with metformin.

**Conclusions:**

Our results suggest that diabetes treatment does not influence the risk of cancer associated with type 2 diabetes. Therefore, an eventual increase of cancer should not be a reason for biasing the selection of any glucose-lowering treatment in type 2 diabetic population.

## Introduction

Type 2 diabetes has been associated with an increased risk of cancer. This population indeed has a greater risk of three of the leading causes of cancer mortality such as pancreatic, colorectal and breast cancer [1]. In addition, type 2 diabetes is associated with substantial premature death rates from several types of cancer [2].

The etiology of this excess cancer risk is poorly understood. Type 2 diabetes and cancer have common risk factors including age, race/ethnicity, obesity, physical inactivity, and tobacco use [1]. Data from large randomized controlled trials of intensified glycemic control suggest that cancer risk is not reduced by improving glycemic control in type 2 diabetes [3], and that both obesity and insulin resistance with or without hyperglycemia are also associated with an increased risk of cancer [4], [5]. Therefore, factors other than glucose could be involved in the relationship between type 2 diabetes and cancer development. Among these factors it seems that hyperinsulinemia and/or insulin resistance could play an essential role. In fact, the presence of insulin resistance and hyperinsulinemia, may accelerate tumor growth [6].

The role of insulin in cancer promotion is suggested by studies associating circulating insulin levels and cancer of the colon, pancreas, and breast [1], [6], [7]. The association between exogenous insulin and cancer gained attention in 2009 when three observational studies evaluating cancer risks with different types of insulin were published concurrently [8]–[10], fuelling speculation of an increased risk of cancer (in particular breast cancer) associated with the insulin analogue insulin glargine, due to its higher affinity for the IGF-1 receptor in comparison with human insulin. More recently, several studies have found a lack of relationship between insulin glargine and overall cancer incidence [11]–[14].

Apart from insulin, other glucose-lowering therapies have been involved in the relationship between type 2 diabetes and cancer. Several observational studies have suggested an increased risk of cancer or cancer mortality with sulfonylureas [15]–[17]. This finding could be explained by sulfonylureas capacity to increase circulating insulin levels. Glucagon-like peptide-1 Receptor (GLP-1R) agonists and dipeptidyl peptidase-4 (DPP-4) inhibitors have also been associated with thyroid and pancreatic cancer [18]. By contrast, metformin [19]–[25] and thiazolidinediones (TZDs) [26]–[28] have been associated with a reduced risk of cancer. This association may be due to the ability of these drugs to reduce insulin resistance.

Given the clear relationship between type 2 diabetes and cancer incidence it seems important to dissect the potential role of any glucose-lowering therapy in the cancer risk. In this regard, the aim of the present study was to evaluate the impact of glucose-lowering agents in the risk of cancer in a large type 2 diabetic population.

## Methods

### Data Source

The information was obtained from the Catalan Institute of Health and electronically fielded by using the System for the Development of Research in Primary Care (SIDIAP) database. This comprises the clinical information coded in the corresponding medical records from 274 Primary Health Care Centers (PHCC) with a total of 3,414 general practitioners and with a global adult (over 15 years old) population of 7,434,632 subjects. The SIDIAP includes data from primary care electronic medical records (demographics, consultations with GPs, diagnoses, clinical variables, prescriptions and referrals), laboratory test results and medications (obtained from CatSalut prescription drug pharmacy invoice database) [29].

For the present study, the validity of the Electronic Health Records (EHR) data was assessed by checking whether the continuous variables were within biologically plausible ranges. The reliability of the EHR data was assessed by assuring the concordance between related variables such as frequency of diabetes diagnosis and frequency of antidiabetic drugs use. In addition, the prevalence of type 2 diabetes and the use of antidiabetic drugs obtained in the 16 PHCC included in the study was within the range reported in our country. Since the SIDIAP database was established, different studies have confirmed the validity of its information [29]–[33].

The diagnosis of type 2 diabetes and cancer was established according the *International Classification of Diseases*, 10^th^ revision (ICD-10) [34]. A case was defined as a patient with cancer diagnosis retrieved from the Hospital Vall d'Hebron Cancer Registry. This is an anonymous specialized registry that includes all patients with cancer diagnosis attending this hospital and collects demographic and clinical data, cancer site, pathology description, cancer diagnosis date (the first ever mention of cancer irrespective of its stage), and death (if applicable).

This clinical investigation was conducted according to the principles expressed in the Declaration of Helsinki. The Study Protocol was approved by the Clinical Research Ethics Committee of Hospital Universitari Vall d'Hebron (Barcelona). This Committee waived the need for written informed consent. All data were anonimyzed and the confidentiality of medical records was respected at all times according to the law (Organic Law 15/1999 on the Protection of Personal Data. http://www.boe.es/boe/dias/1999/12/14/pdfs/A43088-43099.pdf). As this study was a retrospective analysis, there was no need for informed consent.

### Study Cohort

A total of 275,164 type 2 diabetic patients older than 40 years registered in the SIDIAP and attending 16 Primary Health Care Centers (PHCC) of Barcelona were included in the study.

### Case and control selection

A nested case-control study was conducted within the defined cohort following the Strobe rules [35].

Cases (n = 1040) comprised those type 2 diabetes subjects with any cancer [10th Revision (ICD-10) codes version: C00-C97 [34]] diagnosed between 2008 and 2010, registered at the Cancer Registry of Hospital Universitari Vall d'Hebron (Barcelona).

In order to perform the analysis of the present study both databases, SIDIAP and the Hospital Universitary Vall d'Hebron Cancer Registry were linked. Exclusion criteria were: 1) No sufficient elapsed time (<1 year) from diagnosis of diabetes and cancer detection. 2) Less than two visits to the PHCC in the last year. 3) Patients who had received treatment outside of the Catalan Institute of Health service system. 4) Patients in whom the diagnosis of cancer were non-coincident between the Cancer Registry of Hospital Universitari Vall d'Hebron and the SIDIAP database. Controls were obtained from the population with type 2 diabetes registered in SIDIAP (n = 275,164). After excluding those patients with a history of cancer and those with less than two visits at the PHCC, a total of 235,332 possible controls were obtained. Then, three controls for each case were matched by age (+/−5 years), sex, diabetes duration (+/−1.5 years), and geographical area (from the same PHCC).

### Exposure assessment

Data on medication use were obtained from the CatSalut prescription drug pharmacy invoice database. For each case, the treatment received during the 3 years before the diagnosis of cancer was taken into consideration. Treatment with antidiabetic drugs had to have started at least 6 months prior to the diagnosis of cancer. The variable treatment was gathered in 10 groups: insulin glargine, insulin detemir, human insulin, fast-acting insulin and analogues, metformin, sulfonylureas, repaglinide, thiazolidinediones (TZD), DPP-4 inhibitors, and alpha glucosidase inhibitors. For each treatment, exposure time and dose defined as DDD (Daily Dose Defined) divided by total number of days were considered in the analysis.

GLP-1R agonists were not included because they were not licensed in Spain.

### Variables

The information obtained from SIDIAP database included the following variables: gender, age (calculated from birth date), duration of diabetes (calculated from diabetes diagnosis date), BMI (body mass index) at the time of cancer diagnosis, smoking habit (one or more cigarettes per day) classified as former and current smokers versus nonsmokers, and alcohol intake (regular intake of >1 drink per day) classified as yes/no at the time of cancer diagnosis. HbA1c level measured within the 6 months previous to cancer diagnosis and the defined daily dose (DDD) and duration of treatment (months) of glucose-lowering agents were taken into account. Finally, the use of statins and aspirin, two drugs commonly used by type 2 diabetic patients that have been associated with a reduced risk of cancer [36] were also considered.

Information about cancer diagnosis date, tumor location, pathological characteristics, date of death (if applicable) was also collected from the Cancer Registry of Hospital Universitari Vall d'Hebron.

### Statistical analysis

To estimate the sample size, the following proportions of treatments were used: insulin glargine (7.25%), insulin detemir (3.48%), insulin NPH (7.35%), and without pharmacological treatment (22.92%). To estimate the odds ratio (OR) of exposure to differents insulins and oral hypoglycemic agents, we considered the prevalence of insulin detemir because it required a larger sample size. A total of 554 cases and 1662 controls were necessary in order to detect an OR at least of 2 in patients exposed to treatment compared to those not receiving, using a two-tailed significant alpha level of 0.05 and 80% of power. These calculations were performed with PASS 2008 and with EPIDAT, version 3.1.

An initial descriptive analysis was performed using mean (standard deviation) for quantitative variables and frequency (percentage) for qualitative variables. To assess differences between cases and controls Chi-squared or Fisher test for qualitative variables and T-test or Kruskal test for quantitative variables were performed.

We conducted a conditional logistic regression to estimate the crude and adjusted OR (95% CI) of risk of cancer for each treatment group. Since the design of the study is a nested case-control study (1 case and 3 matched controls), a total of 764 groups were considered for the analysis. In each group the case and the 3 controls could be either exposed or non-exposed. In order to calculate the adjusted OR the following confounders were taken into account: age, BMI, alcohol use, smoking and duration of diabetes. In order to take into account the dose and duration of each treatment, two more conditional logistic regression models were performed for each glucose-lowering drug. The main exposure was dose (DDD in tertiles vs. non-users) in the first one and duration (months in tertiles vs. non-users) in the second one. All these models were also adjusted for the same explanatory variables and for the use of other glucose-lowering drugs. The methodology chosen for the final models was the one developed by Hosmew and Lemeshow [37]. The level of statistical significance was 0.05 and all the analysis were performed using IBM SPSS Statistics 19 and R software version 2.10.1.

## Results

Out of 1040 diabetic patients with cancer according to the inclusion criteria, 276 cases were excluded. Among them, 164 were excluded because diabetes mellitus was diagnosed in the year previous to cancer diagnosis; 14 cases did not make at least 2 visits to PHCC during the year previous to cancer diagnosis and finally 98 cases were excluded because the diagnosis of cancer was not well defined. Therefore, a total of 764 patients (34,5% female and 65.5% male) diagnosed with cancer were considered for the study. The number of patients by cancer site is shown in Table 1.

**Table 1:** Number of cases by cancer site included in the study.

Cancer Site	n	%
Colon	108	14.14
Lung	96	12.57
Prostate	96	12.57
Breast	81	10.60
Urinary bladder	80	10.47
Stomach	43	5.63
Uterus	40	5.24
Liver	40	5.24
Oral cavity	36	4.71
Pancreas	24	3.14
Other	120	15.69

**Figure 1:**
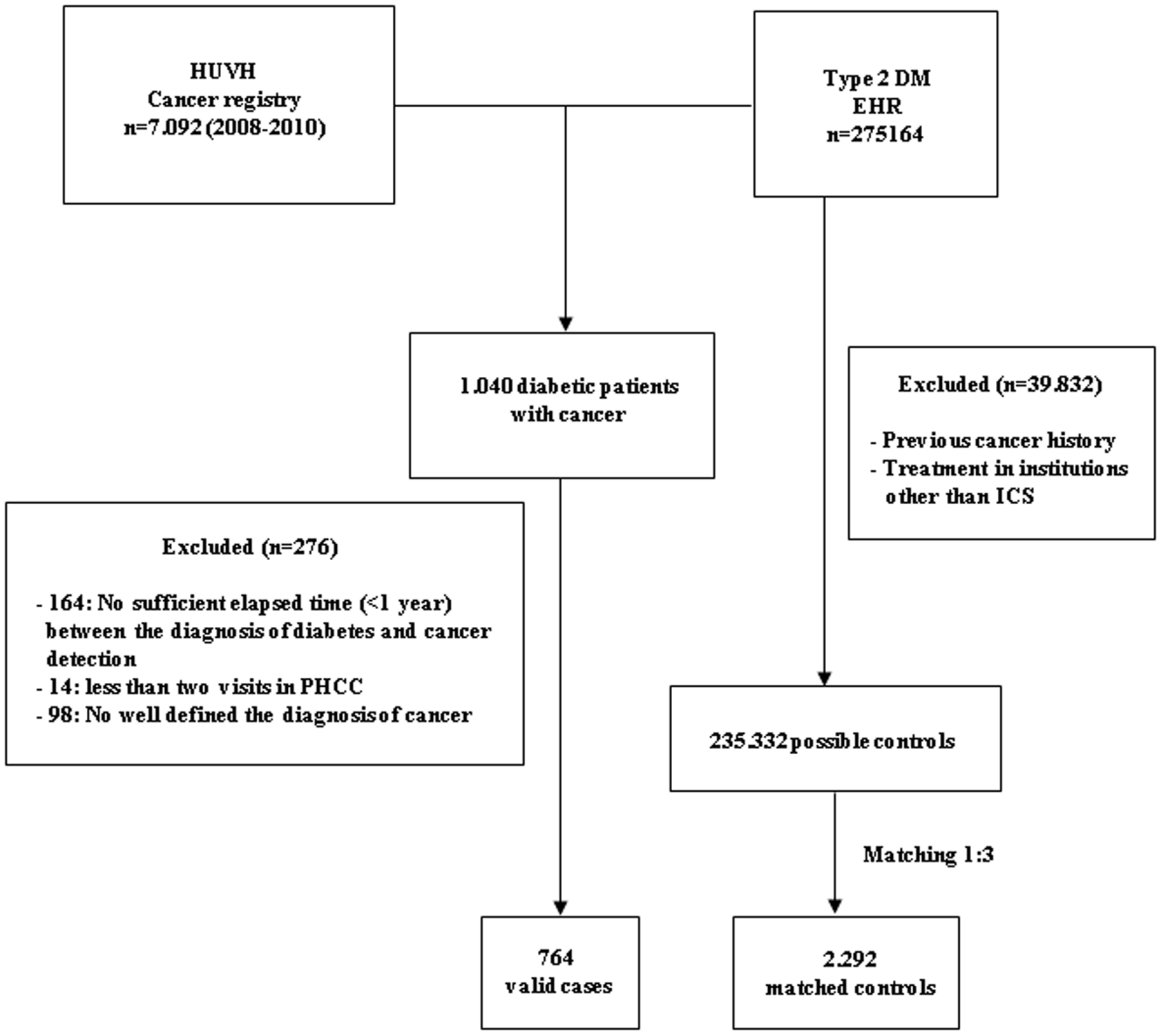
Flow chart of selection of cases and controls. EHR: Electronical Health Record (Barcelona region); HUVH: Hospital Universitari Vall d'Hebron. ICS: Catalan Institute of Health; PHCC: Primary Health Care Centers.

From 235,332 possible controls, 2,292 (34.5% women and 65.5% men) were selected according to similarity with the corresponding assigned case (3 controls for each case). Figure 1 show the flow chart of the selection patient process.

Information regarding cases and controls related to demographic characteristics and treatment received are summarized in Tables 2 and 3. The mean age of cases and controls were 72 (SD 9.1). As reported in Table 2, case subjects and matched controls were similar in age, gender, diabetes duration and HbA1c levels. BMI and smoking habit were higher in cases than in control group but did not reach statistical significance.

**Table 2:** Clinical characteristics of the diabetic population included in the study.

		TOTAL	Cases	Controls	p-value
N of patients		3056	764	2292	
Gender	n(%)	3056 (100)	764 (100)	2292 (100)	
	Male	2000 (65.5)	500 (65.5)	1500 (65.5)	
	Female	1056 (34.5)	264 (34.5)	792 (34.5)	1.000
Age at cancer in years	n (%)	3056 (100)	764 (100)	2292 (100)	
	mean (SD)	72.0 (9.1)	72.0 (9.1)	72.0 (9.1)*	0.975
Age at DM in years	n (%)	3056 (100)	764 (100)	2292 (100)	
	mean (SD)	65.6 (9.5)	65.6 (9.5)	65.6 (9.5)	0.961
Diabetes' duration in years	n (%)	3056 (100)	764 (100)	2292 (100)	
	mean (SD)	6.4 (4.4)	6.4 (4.4)	6.4 (4.4)	0.864
BMI in kg/m2	n (%)	2602 (85.1)	669 (87.6)	1933 (84.3)	
	mean (SD)	29.6 (4.6)	29.3 (4.6)	29.2 (4.6)	0.057
Alcohol	n (%)	3056 (100)	764 (100)	2292 (100)	
	yes	110 (3.6)	31 (4.1)	79 (3.4)	
	no	2946 (96.4)	733 (95.9)	2213 (96.6)	0.433
Tobacco	n (%)	2932 (95.9)	732 (95.8)	2200 (96.0)	
	yes/former	1073 (36.6)	290 (39.6)	783 (35.6)	
	never	1859 (63.4)	442 (60.4)	1417 (64.4)	0.051
HbA1C†	n (%)	2000 (65.4)	522 (68.3)	1478 (64.5)	
	mean (SD)	6.9 (1.4)	6.9 (1.4)	6.9 (1.4)	0.812

* Age at day of cancer of the corresponding case.

† At least into the 6 months previous to cancer diagnosis.

m (SD) = mean and standard deviation.

DM = Diabetes Mellitus type 2.

**Table 3:** Glucose-lowering agents received by the diabetic population included in the study.

		TOTAL	Cases	Controls	p-value
N of any treatment		2438	609	1829	
Insulin Glargine	n(%)	128 (4.2)	31 (4.1)	97 (4.2)	0.917
	Duration months, m(SD)	20 (10.9)	20.1 (12.2)	19.9 (10.5)	0.920
	n° DDD, m(SD)	455.5 (305.9)	475 (309.8)	449.2 (305.9)	0.709
	n°DDD/days, m (SD)	0.84 (0.44)	0.88(0.36)	0.83 (0.46)	0.336
	Units/Kg/day, n°	108	24	84	
	Units/Kg/day, m (SD)	0.44 (0.24)	0.49 (0.21)	0.43 (0.25)	0.128
	Units/Kg/day≥0.3, n(%)	81 (75.0)	21 (87.5)	60 (71.4)	0.180
Human Insulin NPH	n(%)	392 (12.8)	90 (11.8)	302 (13.2)	0.349
	Duration months, m(SD)	27 (10.9)	27.1 (11.5)	26.9 (10.7)	0.668
	n° DDD,m(SD)	781.2 (616.8)	743.9 (497.6)	792.4 (648.4)	0.805
Insulin Detemir	n(%)	98 (3.2)	27 (3.5)	71 (3.1)	0.554
	Duration months, m(SD)	20.2 (10.7)	21.2 (11.3)	19.8 (10.6)	0.588
	n° DDD, m(SD)	567.5 (501.0)	476.4 (318.0)	602.1 (553.1)	0.408
Fast-acting insulin	n(%)	184 (6.0)	53 (6.94)	131 (5.7)	0.220
and analogues	Duration months, m(SD)	27.8 (9.7)	29.5 (8.5)	27.2 (10.1)	0.277
	n° DDD,m(SD)	892.9 (625.6)	999.5 (711.3)	849.8 (584.9)	0.255
Metformin	n(%)	1762 (57.7)	430 (56.3)	1332 (58.1)	0.375
	Duration months, m(SD)	27.9 (10.1)	27.6 (10.4)	28 (10.0)	0.433
	n° DDD, m(SD)	619.7 (386.1)	613.8 (389.2)	621.6 (385.2)	0.648
Sulfonylureas	n(%)	1233 (40.4)	294 (38.5)	939 (41.0)	0.233
	Duration months, m(SD)	28 (9.9)	28.6 (9.5)	27.8 (10.0)	0.221
	n° DDD, m(SD)	1114.2 (909.5)	1086.5 (834.6)	1122.9 (932.0)	0.971
Repaglinide	n(%)	164 (5.4)	40 (5.2)	124 (5.4)	0.926
	Duration months, m(SD)	23.8 (11.5)	22.9 (10.7)	24.1 (11.8)	0.474
	n° DDD, m(SD)	527.7 (466.1)	564.7 (477.9)	515.8 (463.6)	0.799
Thiazolidinediones	n(%)	146 (4.8)	33 (4.3)	113 (4.9)	0.557
	Duration months, m(SD)	22.5 (10.6)	22.1 (11.2)	22.6 (10.5)	0.874
	n° DDD,m(SD)	661.1 (440.5)	619.3 (439.2)	673.3 (442.1)	0.398
DPP-4 Inhibitors	n(%)	75 (2.5)	19 (2.5)	56 (2.4)	1.000
	Duration months,m(SD)	11.8 (6.6)	10.7 (5.3)	12.1 (6.9)	0.591
	n° DDD, m(SD)	367.9 (213.5)	308.7 (164.9)	388.1 (225.4)	0.209
Alpha Glucosidase	n(%)	115 (3.8)	24 (3.1)	91 (4.0)	0.325
Inhibitors	Duration months, m(SD)	23 (12.0)	19.3 (10.6)	24 (12.2)	0.038
	n° DDD, m(SD)	382 (350.3)	263.2 (204.4)	413.4 (374.2)	0.132

m (SD) = mean and standard deviation.

**Figure 2:**
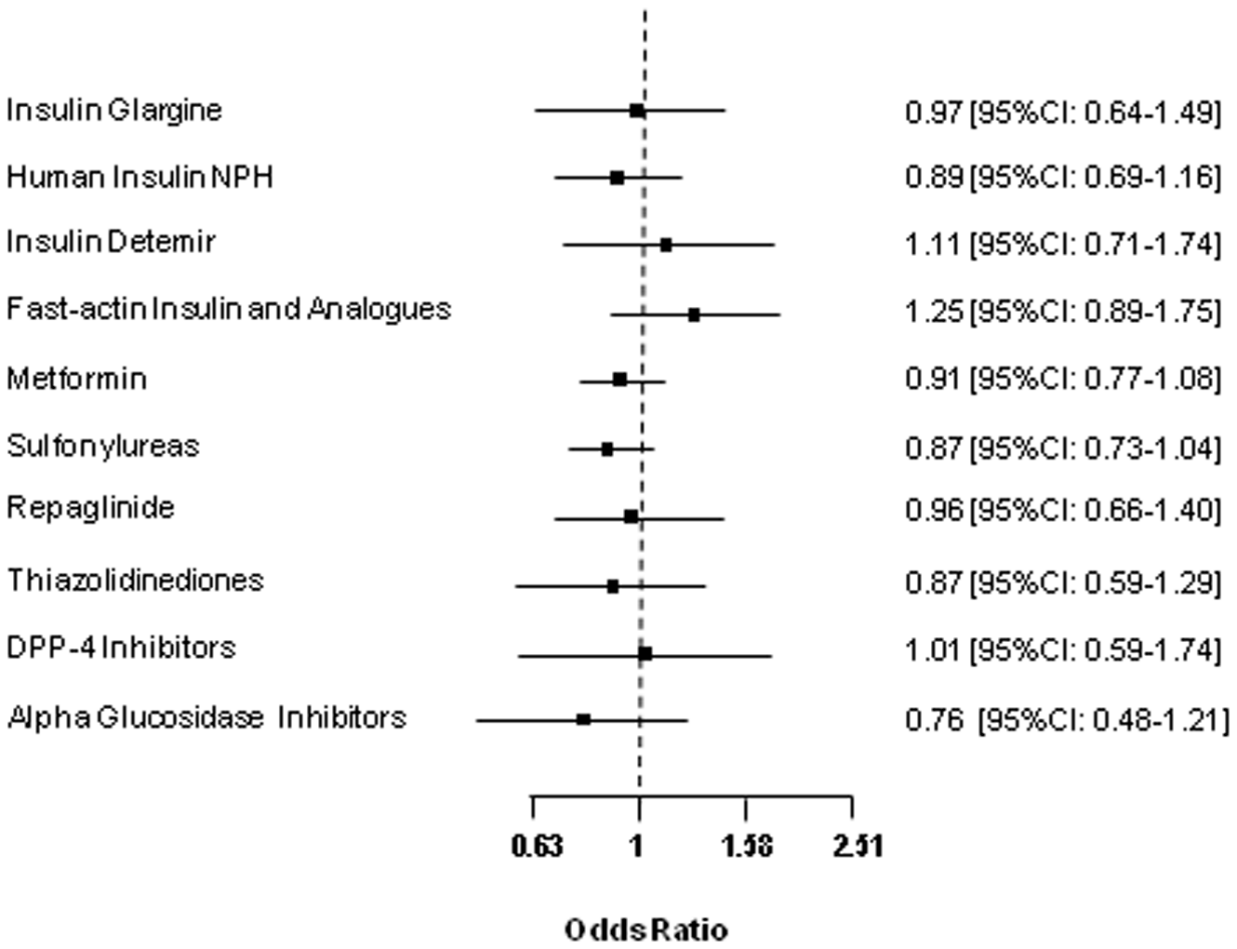
Risk of cancer associated with drugs (use *vs.* non-use). OR [CI95%] adjusted for age, BMI, duration of diabetes, alcohol and tobacco consumption.

Regarding antidiabetic treatment, no statistically significant differences were observed between case and control subjects for the proportion, dose or duration of exposure to each treatment (Table 3). Since a dose higher than 0.3 UI/Kg/day has been associated with an increase of cancer risk [38] we have also considered this cut-off point in the analysis of the results. Figure 2 shows the risk of cancer associated with several types of insulin and oral agents analyzed as “use” versus “non-use”, and adjusted for age at cancer diagnosis, BMI, alcohol consumption, tobacco and duration of diabetes. None of them showed a significant increase in the risk of cancer. Given that no differences between cases and controls in the use of statins and aspirin were observed (data not shown), these variables were not included in the multivariate analysis.

When considering the dose and duration of each glucose-lowering drug, no statistically significant differences were observed. Among those patients who received glargine treatment, the adjusted OR of DDD divided by duration of treatment in days was 0.55 (95% CI = 0.24–1.25) in the first tertile, 1.37 (95% CI 0.70–2.66) in the second tertile and 1.11 (95% CI 0.55–2.25) in the third tertile. The OR of duration of treatment in months among those who received glargine treatment was 1.10 (95% CI = 0.55–2.22), 0.95 (95% CI = 0.55–1.64) and 0.68 (95%CI = 0.15–3.17) in the first, second and third tertiles, respectively. For both analyses ‘no glargine treatment’ was the reference category. Finally, no increased cancer risk was observed when glargine was used alone or in combination with metformin (OR 0.94 [95%CI 0.48–1.86] vs. 0.99 [95%CI 0.58–1.69]).

## Discussion

This study was aimed to evaluate the impact of glucose-lowering agents in the risk of cancer in a large type 2 diabetic population. We have found a lack of relationship between glucose-lowering therapies and cancer incidence.

In recent years, insulin glargine has been suggested to increase the risk of breast cancer in type 2 diabetic patients [8]–[10], [39]. By contrast, metformin seemed to reduce the cancer in the same population of patients [19]–[25], [40]. However, several potential caveats and methodological concerns have been raised in some of this studies including but not limited to cohort definitions, data elements and analytical approach.

The possible relationship between insulin glargine and the increase of cancer risk has been a topic which has generated intense debate in recent years. A significant increased risk for all types of cancer [8], breast cancer in women [9], [10], [12], and prostate and pancreatic cancer in men [11] has been reported in some studies. Conversely, some others have found a neutral effect [11]–[14] or even a significant protection for overall cancer incidence [38]. In our study, we did not find any increase of risk of cancer associated with insulin glargine with a mean exposition time of 2 years. Our results agree with the recent French nationwide cohort study based on administrative databases with a median follow-up of 2.67 years [41], and the ORIGIN trial with a prospective follow up of up to 7 years [42]. Moreover, we did not find any relationship between doses of insulin glargine and risk of cancer.

Insulin glargine is a recombinant DNA analog of human insulin with three amino acid substitutions: two arginine residues are added to the chain B at positions 31 and 32 and glycine is substituted for histidine at position 21 in the A chain. These amino acid substitutions result in an increased binding affinity for the insulin and insulin-like growth factor-1 (IGF-1) receptors, six to eight times that of human insulin [42]. *In vitro* studies have shown that glargine is more mitogenic than human insulin and promotes proliferation of certain tumor cells [43]. However, this mitogenic activity has been shown exclusively at supraphysiological concentrations (nanomolar/micromolar) and it is related to the expression of the IGF-1 receptor; the effect being present in cells with high levels of the receptor and absent in cells with limited or no IGF-1 receptor expression. In animal studies, glargine did not promote tumor growth, despite being administered at these supraphysiological concentrations, which are unlikely to be achieved in the clinical practice as the doses needed to produce these concentrations are liable to lead to hypoglycemia. Furthermore, glargine in *in vivo* experiments is rapidly transformed into its metabolites, the metabolic and mitogenic characteristics of which have been shown to be broadly equal to or even lower than human insulin [44]. Finally, the concentration of insulin glargine reaching the tumoral cells is unknown but, as can be deduced, much lower than that used *in vitro*. Thus, the suggestion of increased relative mitogenic potency of insulin glargine seen in some cell lines does not appear to carry over to the *in vivo* behavior in animals and humans.

A small number of studies found a higher risk of cancer or cancer death among individuals with diabetes who were treated with sulfonylureas compared with those treated with metformin or other anti-diabetic drugs [15]–[17]. However, a recent meta-analysis did not find that sulfonylurea affected the risk of any type of cancer [45].

Several studies have suggested that both metformin [19]–[25] and TZD [26]–[28] are associated with a reduced risk of cancer in diabetic subjects. Given the biologically plausible link between diabetes and cancer, mediated through insulin resistance and hyperinsulinemia, this effect may be due to the ability of these drugs to reduce insulin resistance, although there may also be specific cellular mechanisms, mediated in part through AMP-activated protein kinase (AMPK) signaling pathways [46]. However, a recent meta-analysis failed to confirm this protective action of metformin [47] and even an increased risk of bladder cancer has been associated with TZDs [48]. We did not find that either metformin or TZDs had any effect on the risk of cancer in our type 2 diabetic population.

Incretin mimetics have been associated with an increased risk of pancreatic cancer [18]. However, this association was been based on the Food Drug Administration Adverse Event Reporting System (FAERS) database which cannot be considered an acceptable registry to compare adverse event rates between drugs because of reporting biases and incomplete data. We found a lack of relationship between DDP-4 inhibitors and the risk of cancer.

Most of the published results on the potential influence of glucose-lowering treatments and cancer incidence have been drawn from observational studies with different approaches. This is considered to be the main reason for the controversial results between the studies. In addition, there are several limitations in the clinical assessment in these observational studies including the failure to correct for BMI, the duration of diabetes, the dosage of glucose-lowering treatments, the impossibility of breaking down the risk of cancer from a general to a tumor-specific risk, and the lack of information on tobacco use. In our study, we have considered all these confounding factors and we have not found any effect of glucose-lowering agents on the risk of cancer. In addition, all the patients included in the study were periodically followed by physicians and nurses working for the same health-care provider (*Catalan Health Institute*) using similar protocols and the same electronic clinical record registry. Furthermore, a central Cancer Registry was used. This is worth mentioning because 98 out of 1,040 diabetic patients were excluded because of discrepancies between the clinical record and the cancer registry. Another point to be commented on is the so called “protopathic bias”. This is the bias towards the use of more potent antidiabetic agents due to the poor glycemic control secondary to the presence of a hidden cancer. In this regard, the protopathic bias has been reported as the main factor accounting for the increased risk of cancer in the first 6 months after starting treatment with insulin and sulfonylureas [49]. In the present study the 6 month window for antidiabetic treatment prior to cancer diagnosis seems a sufficient period to significantly reduce any eventual protopathic bias. Finally, a potential bias due to the use of statins and aspirin, two drugs commonly used by type 2 diabetic patients that exhibit an antineoplasic effect [36] can be ruled out because we did not find any differences between cases and controls in the use of these drugs.

This study has several limiting factors: 1) The information regarding glucose lowering treatments in HER was only available since 2005, and, therefore, we have no information on the occurrence of cancers after long-term exposure. 2) Our analysis only attributed a case of cancer when this event occurred at least 6 months after the start of exposure, a period that could be considered acceptable by exploring the antidiabetic drugs with an eventual oncogenic effect but probably short for drugs with potential protective effects like metformin. 3) Specific information on the effect of glucose-lowering agents by cancer site is not provided because the low number of cancers per site prevented us to performing a valid statistical analysis. 4) Another problem is that a high number of patients were taking more than one antidiabetic drug, thus making it very difficult to dissect the cancer risk attributed to an individual treatment [50]. In addition, the comparator group for each antidiabetic drug was composed of the patients taking the remaining antidiabetic drugs. Ideally, the clinical effect of each antidiabetic drug should be evaluated by analyzing only diabetic patients with monotherapy and comparing them with those with no pharmacological therapy (only diet). However, this ideal scenario can hardly be achieved because a high percentage of type 2 diabetic patients are not in monotherapy, and treatments based only on diet are not now currently recommended. In fact, few studies comparing the incidence of cancer with antidiabetic drugs in monotheraphy have been reported [35]. 5) Another limiting factor is the the absence of available data on GLP-1 R agonists. These drugs were not included in data analyses because they were recently introduced into our country at study entry, thus resulting in a very low number of cases. 6) Finally, we rely on doses dispensed rather than dose administered, a reasonable but imperfect measure of real use.

In conclusion, our results suggest that anti-diabetic treatment does not influence the risk of cancer associated with type 2 diabetes. Therefore, an eventual increase of cancer should not be a reason for biasing the selection of any glucose-lowering treatment in type 2 diabetic population.
